# HV-LIOM: Adaptive Hash-Voxel LiDAR–Inertial SLAM with Multi-Resolution Relocalization and Reinforcement Learning for Autonomous Exploration

**DOI:** 10.3390/s25247558

**Published:** 2025-12-12

**Authors:** Shicheng Fan, Xiaopeng Chen, Weimin Zhang, Peng Xu, Zhengqing Zuo, Xinyan Tan, Xiaohai He, Chandan Sheikder, Meijun Guo, Chengxiang Li

**Affiliations:** 1 School of Mechatronical Engineering, Beijing Institute of Technology, Beijing 100081, China; 3120230131@bit.edu.cn (S.F.); zhwm@bit.edu.cn (W.Z.); 3120220159@bit.edu.cn (P.X.); 3120215092@bit.edu.cn (Z.Z.); 3220225052@bit.edu.cn (X.T.); 3120235415@bit.edu.cn (X.H.); chandan@bit.edu.cn (C.S.); 3220230244@bit.edu.cn (M.G.); 3120230134@bit.edu.cn (C.L.); 2 Zhengzhou Research Institute, Beijing Institute of Technology, Zhengzhou 450000, China

**Keywords:** LiDAR-based SLAM, Hybrid Voxel Mapping, active exploration, reinforcement learning, autonomous exploration

## Abstract

This paper presents HV-LIOM (Adaptive Hash-Voxel LiDAR–Inertial Odometry and Mapping), a unified LiDAR–inertial SLAM and autonomous exploration framework for real-time 3D mapping in dynamic, GNSS-denied environments. We propose an adaptive hash-voxel mapping scheme that improves memory efficiency and real-time state estimation by subdividing voxels according to local geometric complexity and point density. To enhance robustness to poor initialization, we introduce a multi-resolution relocalization strategy that enables reliable localization against a prior map under large initial pose errors. A learning-based loop-closure module further detects revisited places and injects global constraints, while global pose-graph optimization maintains long-term map consistency. For autonomous exploration, we integrate a Soft Actor–Critic (SAC) policy that selects informative navigation targets online, improving exploration efficiency in unknown scenes. We evaluate HV-LIOM on public datasets (Hilti and NCLT) and a custom mobile robot platform. Results show that HV-LIOM improves absolute pose accuracy by up to 15.2% over FAST-LIO2 in indoor settings and by 7.6% in large-scale outdoor scenarios. The learned exploration policy achieves comparable or superior area coverage with reduced travel distance and exploration time relative to sampling-based and learning-based baselines.

## 1. Introduction

Autonomous 3D mapping and localization are fundamental capabilities for mobile robots operating in complex, GNSS-denied environments. With the rapid advances in artificial intelligence and embodied intelligence, mobile robots are increasingly deployed in indoor inspection, environmental exploration, disaster response, and autonomous delivery. Their effectiveness relies critically on the accuracy, robustness, and computational efficiency of simultaneous localization and mapping (SLAM), which enables perception, mapping, and navigation without external positioning infrastructure.

Recent LiDAR–inertial SLAM systems have achieved impressive real-time performance by combining efficient point-cloud registration, tightly coupled LiDAR–IMU fusion, and global optimization. Representative methods such as LOAM [1], LIO-SAM [2], FAST-LIO2 [3], and their variants demonstrate that feature-based or direct registration on k-d tree or voxel-grid maps, together with optimization- or filtering-based fusion, can provide accurate and robust localization across diverse indoor and outdoor scenarios. Despite this progress, several limitations remain. First, map representations are often built upon static k-d tree or octree structures, leading to high memory consumption and degraded query/update efficiency as the map grows. Second, multi-session capabilities—including prior-map relocalization and long-term reuse of previously built maps—are frequently implemented as separate add-ons or not explicitly addressed. Third, autonomous exploration is commonly driven by handcrafted heuristics, lacking a unified learning-based decision-making module that is tightly coupled with the SLAM backbone.

To address these limitations, we propose HV-LIOM, a unified LiDAR–inertial SLAM and autonomous exploration framework that integrates adaptive hash-voxel mapping, learning-based loop closure, multi-resolution prior-map relocalization, and a deep reinforcement learning policy for active exploration. We refer to the overall SLAM and exploration system as HV-LIOM (Adaptive Hash-Voxel LiDAR–Inertial Odometry and Mapping). The LiDAR–inertial odometry front-end is denoted *HV-LIO*, and the full SLAM system with loop closure and global optimization is denoted *HV-LIO-SAM*. Unlike conventional LiDAR–inertial pipelines that primarily emphasize odometry and static mapping, HV-LIOM explicitly targets long-term, multi-session operation and autonomous exploration in unknown environments, while preserving real-time performance and high localization accuracy.

In summary, our contributions are as follows:

- We propose an adaptive hybrid hash-voxel mapping strategy that models both planar and non-planar structures. The representation adaptively maintains surface and non-surface features, enabling efficient feature association and high-fidelity incremental map updates with improved memory efficiency.
- We develop a learning-based loop-closure mechanism based on the Boundary Triangle Context (BTC) descriptor, which encodes geometric similarity between keyframes via triangle-level relational encoding. Integrated with global factor-graph optimization, this module improves global consistency and mitigates long-term drift.
- We design an active exploration strategy based on the Soft Actor–Critic (SAC) algorithm, enabling robots to select informative navigation targets by balancing information gain and motion cost. This learning-based policy improves the efficiency and completeness of exploration in unknown environments.
- We integrate a multi-resolution Normal Distributions Transform (NDT) relocalization module that performs coarse-to-fine probabilistic alignment, providing robust initialization and accurate localization against prior maps across diverse deployment conditions.

## 2. Related Work

### 2.1. LiDAR–Inertial SLAM

Point cloud registration is a central component of LiDAR–inertial SLAM [4,5]. Since the seminal Iterative Closest Point (ICP) algorithm [6], LiDAR-based SLAM has achieved substantial progress in registration accuracy and robustness [7]. However, the sparsity and non-uniformity of spinning LiDAR returns often limit the reliability of direct frame-to-frame ICP, especially in low-structure scenes.

To improve data association under sparse measurements, LOAM [1] extracts edge and planar features and formulates point-to-line and point-to-plane constraints. By combining high-rate odometry with low-rate mapping optimization, LOAM achieves a practical trade-off between accuracy and real-time performance. Building on this paradigm, LeGO-LOAM [8] enhances ground segmentation for wheeled platforms, while LIO-SAM [2] adopts a keyframe-based nonlinear factor-graph formulation to improve global consistency. Extending to solid-state sensors, LOAM-Livox [9] introduces adaptive feature extraction and motion distortion compensation to accommodate non-repetitive scanning patterns.

From the perspective of fusion architectures, loosely coupled approaches based on extended Kalman filtering (EKF) [10,11] are computationally attractive but can degrade in degenerate or low-observability environments. Tightly coupled methods have therefore become prevalent by directly incorporating raw LiDAR and IMU measurements within a unified estimation framework. Optimization-based systems such as LIO-Mapping [12] and IN2LAAMA [13] can achieve high accuracy at the cost of increased computation, whereas filtering-based approaches such as LINS [14] and the FAST-LIO series [3,15] leverage iterative error-state Kalman filtering and efficient map structures to balance precision and efficiency.

Beyond classical geometric feature extraction, recent studies have investigated voxelized representations and learning-based feature encoding to enhance 3D perception and registration. Yuan et al. [16] introduced a multi-hierarchical mutual information learning framework for unsupervised point cloud registration to capture cross-scale geometric dependencies. Cao et al. [17] proposed complementary pseudo-multimodal feature learning for point cloud anomaly detection, demonstrating effective cross-modal fusion. Zhao et al. [18] developed a voxel-pillar multi-frame cross-attention network for robust single-object tracking in sparse point clouds, highlighting the advantages of voxelized spatial encoding for temporal association. These works collectively indicate a shift toward voxelized, learning-informed representations that improve scalability and robustness, which motivates the adaptive hybrid hash-voxel mapping strategy proposed in this paper.

### 2.2. Loop Closure and Global Optimization

During long-term SLAM operation, measurement noise, modeling errors, and unobservable modes can lead to gradual drift accumulation, causing the estimated trajectory to deviate from the true robot motion. As a result, even when the robot revisits a previously mapped place, the trajectory may not form a consistent loop, yielding geometric inconsistencies in the reconstructed map. Loop closure aims to detect such revisits and introduce global constraints, enabling back-end optimization to suppress accumulated errors and maintain global consistency of both the trajectory and the map.

Existing loop closure methods can be broadly categorized into vision-based and point-cloud-based approaches. Vision-based techniques benefit from rich appearance and semantic cues but are sensitive to illumination and viewpoint changes [19]. In contrast, point-cloud-based methods are invariant to illumination and thus more suitable for outdoor and long-term deployment. Early point-cloud approaches such as 3D SIFT [20] and SHOT [21] rely on local descriptors computed from 3D point clouds or projected range images, but their performance can degrade under sparse, non-uniform, or partially occluded measurements. To improve robustness, a series of global descriptors have been proposed, including M2DP [22], Scan Context [23], MinkLoc3D [24], BoW3D [25], Ring++ [26], and STD [27]. More recently, deep learning-based place recognition and loop detection networks such as LCDNet [28], Logg3D-Net [29], LocNet [30], and OverlapNet [31] have further improved accuracy and recall by learning discriminative global and local geometric representations directly from point clouds.

After loop candidates are identified, most LiDAR–inertial SLAM systems employ pose-graph or factor-graph optimization to fuse multiple constraints, including loop closure, inter-frame LiDAR registration, and IMU pre-integration [32]. These constraints are typically formulated as a nonlinear least-squares problem and solved efficiently using incremental smoothing methods such as iSAM2. The optimized global trajectory is subsequently used to update the global map to achieve long-term consistency.

Despite this progress, several challenges remain. Local feature-based methods are sensitive to variations in point density and viewpoint, global descriptors may be confused by repetitive or self-similar structures, and deep learning-based approaches often require large-scale training data and may exhibit limited cross-domain generalization. Therefore, achieving a practical balance among computational efficiency, robustness, and generalization remains a key research direction for loop closure and global optimization in modern SLAM systems.

### 2.3. Active Exploration in Unknown Environments

Traditional SLAM deployments often rely on manual guidance or pre-scripted routes to complete environmental mapping [33,34,35]. To improve robot autonomy, *active exploration* has been widely studied, where robots autonomously plan actions to expand perceptual coverage and reduce mapping uncertainty during online operation.

Existing exploration methods are commonly categorized as frontier-based and sampling-based. Frontier-based exploration, first introduced by Yamauchi [36], selects navigation goals along the boundary between known free space and unknown regions. Subsequent work has improved frontier extraction and decision efficiency through refined segmentation and adaptive goal selection mechanisms [37,38,39]. In contrast, sampling-based methods, exemplified by Next Best View Planning (NBVP) [40], generate candidate viewpoints via randomized tree sampling and choose actions that maximize information gain. Extensions such as TMRRT [41] and GBP [42] further improve real-time performance and long-range exploration capability. However, both paradigms are typically driven by myopic or greedy utility maximization, making it difficult to obtain globally efficient exploration trajectories in large-scale or highly cluttered environments.

To mitigate these limitations, hybrid approaches that combine frontier-based and sampling-based strategies have been proposed. For example, DSVP [43] and TARE [44] adopt hierarchical frameworks to balance local responsiveness and global coverage, improving exploration efficiency and completeness at the cost of increased computational complexity.

In recent years, advances in deep learning and reinforcement learning have motivated data-driven exploration. Some methods employ deep reinforcement learning to adaptively select exploration frontiers [45], whereas others leverage convolutional or graph neural networks to predict information-gain distributions [46]. More recent approaches directly output the next exploration target using end-to-end neural policies [47,48,49,50,51]. While these learning-based methods show promising potential to acquire global exploration priors from data, two challenges remain. First, temporal dependency is often under-modeled, despite active exploration being naturally formulated as a Partially Observable Markov Decision Process (POMDP). Second, reward designs are frequently simplistic or task-specific, which can limit learning efficiency and cross-scene generalization.

### 2.4. Relocalization and Prior Map-Based Localization

Most LiDAR–inertial SLAM systems initialize mapping with respect to the robot’s starting pose and achieve localization through incremental map construction. In practical deployments, however, environments are often pre-mapped, making repeated reconstruction unnecessary. In such cases, the robot can directly localize against a prior map, reducing computational cost and enabling consistent multi-session operation.

Existing relocalization methods can be broadly categorized into point-cloud-registration-based and deep-learning-based approaches. In the first category, classical methods such as ICP and NDT [52] remain widely adopted, while GICP [53] improves accuracy and robustness by better accounting for local surface structure. For NDT-based localization, several extensions have been developed, including 3D-NDT [54], road-marking-aided NDT [55], and Monte Carlo-based NDT [56], which enhance robustness under dynamic or partially changing conditions.

The second category focuses on deep learning-based localization. LocNet [30] learns rotation-invariant features and integrates them with particle filtering for robust matching. DeepLocalization [57] employs dual networks to directly regress the six-degree-of-freedom (6-DoF) pose, while DenserNet [58] and DeLS-3D [59] incorporate multi-scale feature extraction and temporal modeling to further improve registration accuracy. Despite the advantages of parallel inference and data-driven adaptability, learning-based methods often incur substantial computational overhead and rely on large, diverse training datasets, which can hinder generalization and large-scale deployment in real-world settings [60].

To better position HV-LIOM within the literature, we provide a structured comparison of representative LiDAR–inertial SLAM and autonomous exploration methods in Table A1 (Appendix A). The table summarizes key aspects of each approach, including map representation, loop-closure strategy, prior-map relocalization capability, and support for learning-based exploration.

## 3. Methodology

**Figure 1:**
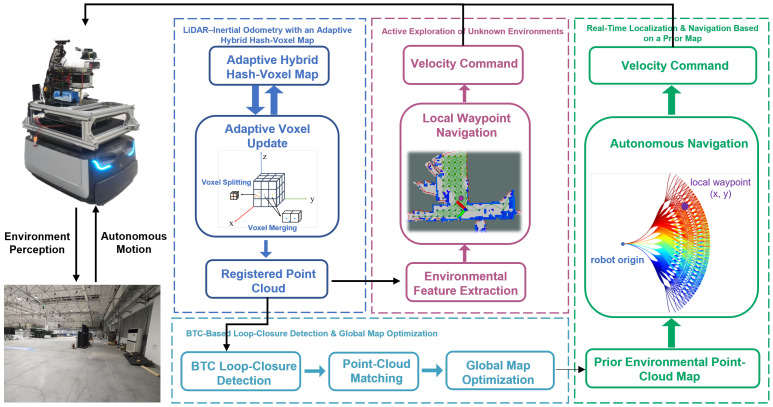
Overall framework of the proposed unified LiDAR–inertial SLAM and active exploration system. The system integrates four main modules: (1) LiDAR–Inertial Odometry with an Adaptive Hybrid Hash-Voxel Map for real-time pose estimation and incremental mapping; (2) BTC-based Loop-Closure Detection and Global Map Optimization for drift correction and global consistency; (3) SAC-based Active Exploration of Unknown Environments enabling autonomous decision-making via spatiotemporal feature learning; and (4) Prior Map-based Real-Time Localization and Navigation for precise relocalization and trajectory planning. All components are deployed on the self-developed mobile robot platform equipped with LiDAR, IMU, and onboard computing units.

This paper presents a unified framework for real-time LiDAR–inertial SLAM and active exploration in unknown environments. As illustrated in Figure 1, the proposed system integrates four key modules to achieve accurate, robust, and autonomous 3D mapping: (i) an adaptive hybrid hash-voxel representation for efficient map management and structural feature modeling; (ii) a BTC-based loop-closure and global graph-optimization module to enforce long-term trajectory consistency; (iii) a SAC-based active exploration policy with spatiotemporal feature modeling for autonomous decision-making; and (iv) a coarse-to-fine prior-map relocalization mechanism that enables reliable localization reuse across multiple mapping sessions.

### 3.1. Adaptive Hybrid Hash-Voxel Map Construction

**Figure 2:**
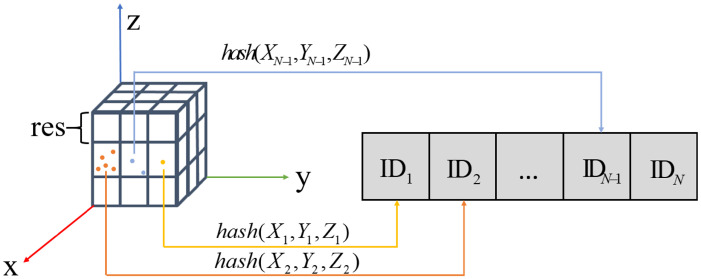
Structure of the Adaptive Hybrid Hash-Voxel Map.

To overcome the limitations of conventional map representations such as k-d trees and octrees, we propose an adaptive hybrid hash-voxel structure. The core idea is to allocate memory only for observed spatial regions via hash indexing while dynamically subdividing or merging voxels according to local point density. As shown in Figure 2, empty space is naturally ignored, and only occupied voxels are stored in a hash table. Each occupied voxel is discretely indexed by its 3D location, enabling efficient correspondence between voxelized map space and hash-table entries for rapid voxel lookup and insertion.

Following common practice in voxel hashing and spatial hashing for 3D reconstruction and real-time mapping [61,62], we adopt a spatial hashing scheme to map 3D LiDAR points into a discrete hash-voxel space.

Specifically, for an observed LiDAR point $p={\left(x,y,z\right)}^{T}$, its discrete voxel coordinate $P={\left(X,Y,Z\right)}^{T}$ is computed from the predefined resolution res as

(1) $$P={\left(X,Y,Z\right)}^{T}={\left(\mathrm{int}\;\left(\frac{x}{res}\right),\mathrm{int}\;\left(\frac{y}{res}\right),\mathrm{int}\;\left(\frac{z}{res}\right)\right)}^{T},$$

where int(·) denotes integer rounding. The corresponding hash-table index ID is then computed as

(2) $$ID=hash\left(P\right)=hash\left(X,Y,Z\right)=\left(\left(X\cdot n_{1}\right)⊕\left(Y\cdot n_{2}\right)⊕\left(Z\cdot n_{3}\right)\right)\;\mathrm{mod}\;N,$$

where $n_{1}$, $n_{2}$, and $n_{3}$ are large prime numbers, *N* is the maximum hash-table capacity, ⊕ denotes bitwise exclusive OR, and mod is the modulo operation with respect to *N*.

This mapping establishes a deterministic point-to-voxel correspondence for efficient feature association and incremental map updates. When LiDAR–inertial odometry performs data association or integrates a newly registered scan, the hash-voxel structure enables rapid retrieval of the relevant voxel statistics and structural parameters.

To further improve association efficiency, each voxel stores the covariance of the associated measurements and fits local structural primitives. Each voxel thus maintains a feature type and the corresponding geometric parameters (e.g., planar or non-planar). Compared with k-d-tree-based LiDAR–inertial SLAM systems such as LIO-SAM and FAST-LIO2 [3], the proposed representation reduces repeated nearest-neighbor queries and per-frame feature fitting. Instead, the odometry front-end directly retrieves pre-stored structural parameters from the matched voxels, thereby improving the efficiency of state estimation and feature association.

**Figure 3:**
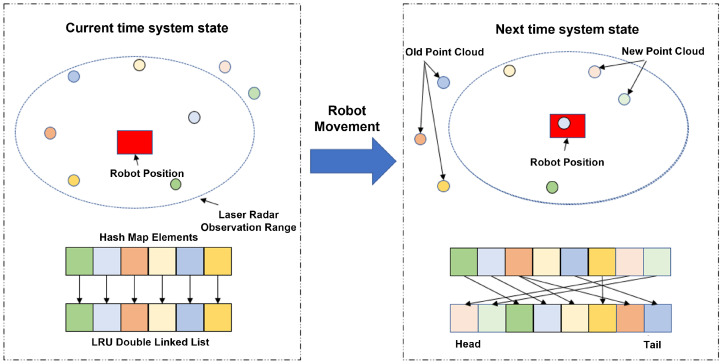
Dynamic maintenance algorithm for the adaptive hybrid hash-voxel map.

Long-term operation on memory-constrained platforms can cause unbounded map growth if not properly controlled. To address this issue, we design a dynamic maintenance strategy for the adaptive hybrid hash-voxel map (Figure 3) to keep memory usage bounded over extended trajectories. In addition to a fixed-capacity hash table, the map maintains a doubly linked list that records voxel IDs. When a new scan is registered after state estimation, the corresponding voxel IDs are inserted at the head of the list. If the number of nodes exceeds a preset capacity, the algorithm removes the tail node and evicts the associated voxels from the hash table. This mechanism effectively limits the number of maintained voxels and enables continuous operation without exceeding memory constraints.

In the current implementation, HV-LIOM assumes quasi-static environments during mapping and localization, consistent with most LiDAR–inertial SLAM frameworks. Transient moving objects are treated as measurement outliers and are filtered using statistical criteria.

### 3.2. BTC-Based Loop Closure and Global Optimization

To mitigate cumulative drift in LiDAR–inertial SLAM, we incorporate a loop-closure mechanism into the back-end optimization. We design a compact binary descriptor, termed the BTC descriptor, to encode local geometric structures into concise binary vectors. The BTC-based loop detector aims to determine whether the current LiDAR frame (frame *j*) corresponds to a previously visited keyframe (frame *i*). Once a match is identified, a loop pair (i,j)∈C is formed, and the relative transformation $\Delta {\tilde{T}}_{i,j}$ is estimated.

**Figure 4:**
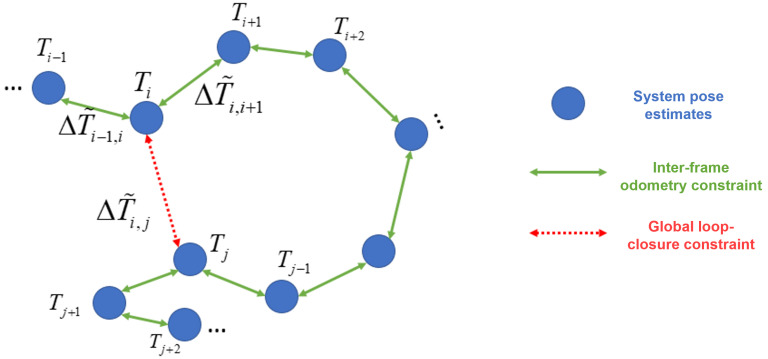
Pose-graph optimization constraints.

Global map optimization seeks a globally consistent pose sequence across *N* time steps,

(3) $$T=\{T_{1},T_{2},\ldots ,T_{N}\},$$

such that all registered point clouds can be aligned in a unified world coordinate system. As illustrated in Figure 4, each node in the pose graph corresponds to a LiDAR–inertial odometry (LIO) pose, whereas edges represent local odometry constraints or global loop-closure constraints.

Following standard formulations in graph-based SLAM and factor-graph optimization [63,64], we model global pose refinement as a nonlinear least-squares problem over odometry and loop-closure constraints.

(4) $$T=\arg\min_{\left\{T_{1},\ldots ,T_{N}\right\}}\left(\sum_{i=1}^{N-1}∥e_{i}{∥}_{\Sigma_{i}^{-1}}^{2}+\sum_{(i,j)\in C}{∥e_{ij}∥}_{\Sigma_{ij}^{-1}}^{2}\right),$$

where $e_{i}$ denotes the relative odometry constraint between adjacent frames and $e_{ij}$ represents the loop-closure constraint between nonadjacent keyframes.

The odometry constraint enforces consistency between consecutive poses.

(5) $$\begin{array}{cc} e_{i} & =\mathrm{Log}\;\left(\Delta {\tilde{T}}_{i,i+1}^{-1}T_{i}^{-1}T_{i+1}\right), \end{array}$$

(6) $$\begin{array}{cc} \Delta {\tilde{T}}_{i,i+1} & ={\tilde{T}}_{i}^{-1}{\tilde{T}}_{i+1}, \end{array}$$

where ${\tilde{T}}_{i}$ and ${\tilde{T}}_{i+1}$ are the pre-optimization LIO estimates.

Similarly, the loop-closure constraint ensures geometric consistency between loop pairs.

(7) $$e_{ij}=\mathrm{Log}\;\left(\Delta {\tilde{T}}_{i,j}^{-1}T_{i}^{-1}T_{j}\right),$$

where $\Delta {\tilde{T}}_{i,j}$ is the relative pose estimated by the BTC-based loop-closure module. The pose-graph objective in (4) and the SE(3) logarithmic error terms in (5)–(7) follow widely used formulations in graph-based SLAM and factor-graph optimization [63,64].

Because odometry and loop-closure constraints typically exhibit different uncertainty levels, their relative weights should be properly modeled. Following first-order error propagation for nonlinear transformations [63,64], the covariance of the odometry constraint is approximated as

(8) $$\Sigma_{i}=\frac{\partial \Delta {\tilde{T}}_{i,i+1}}{\partial T_{i}}\Sigma_{T_{i}}{\left(\frac{\partial \Delta {\tilde{T}}_{i,i+1}}{\partial T_{i}}\right)}^{\;T}+\frac{\partial \Delta {\tilde{T}}_{i,i+1}}{\partial T_{i+1}}\Sigma_{T_{i+1}}{\left(\frac{\partial \Delta {\tilde{T}}_{i,i+1}}{\partial T_{i+1}}\right)}^{\;T},$$

where $\Sigma_{T_{i}}$ and $\Sigma_{T_{i+1}}$ are the pose covariances output by the LIO front-end at time *i* and i+1, respectively.

We further model the loop-closure covariance using the registration confidence produced by the BTC-based fine-alignment module. Specifically, the information matrix is defined as

(9) $$\Sigma_{ij}^{-1}=\alpha \cdot score_{ij}\;I_{6\times 6},$$

where $score_{ij}$ is the loop-closure confidence score, α balances the influence of odometry and loop-closure constraints, and $I_{6\times 6}$ is the 6×6 identity matrix.

#### BTC Descriptor Matching

For each BTC descriptor $C_{i}$ extracted from the current LiDAR frame, its global code $l_{i}$ is used to query the corresponding hash bucket, denoted as $\mathrm{Hash}(l_{i})$, to retrieve candidate descriptors $\left\{C_{j}\right\}$ from the database. Following the BTC-based loop-closure framework in [65], the similarity between a query descriptor $C_{i}$ and a candidate descriptor $C_{j}$ is computed from their local binary encodings $b_{i}=\left\{b_{i}^{1},b_{i}^{2},b_{i}^{3}\right\}$ and $b_{j}=\left\{b_{j}^{1},b_{j}^{2},b_{j}^{3}\right\}$ as

(10) $$\begin{array}{cc} S_{btc}\left(C_{i},C_{j}\right) & =\frac{1}{3}\sum_{k=1}^{3}S_{bin}\left(b_{i}^{k},b_{j}^{k}\right), \end{array}$$

(11) $$\begin{array}{cc} S_{bin}\left(b_{i}^{k},b_{j}^{k}\right) & =\frac{2\cdot HW(b_{i}^{k}\;\&\;b_{j}^{k})}{HW\left(b_{i}^{k}\right)+HW\left(b_{j}^{k}\right)}, \end{array}$$

where HW(·) denotes the Hamming weight and & represents the bitwise AND operation. If $S_{btc}\left(C_{i},C_{j}\right)$ exceeds a predefined threshold, the two descriptors are considered a successful match, and the keyframe ID associated with $C_{j}$ receives a score increment.

### 3.3. SAC-Based Active Exploration

To enable efficient autonomous exploration in unknown environments, we develop an active exploration algorithm based on the Soft Actor–Critic (SAC) framework. The proposed method integrates a topological representation of the local navigation space with spatiotemporal decision features, allowing the robot to learn exploration strategies online through reinforcement learning. A Transformer-based policy network [66] is employed to extract high-dimensional state representations from the topology graph and to output the next best exploration target.

In the SAC updates, we use learning rates $\left(\eta_{Q},\eta_{\pi }\right)$, discount factor γ, and the entropy temperature α. To avoid ambiguity, the bare symbol α is reserved for the SAC entropy temperature, while reward weights always appear with subscripts $\left(\alpha_{e},\alpha_{n},\alpha_{b},\alpha_{d}\right)$.

#### 3.3.1. State Space Design

The state space encodes environmental information relative to the robot. Directly using dense 3D LiDAR point clouds or bird’s-eye-view (BEV) images is computationally expensive. We therefore adopt a navigation topological graph for compact state representation. From the local navigation map, a set of viewpoints $v_{i}=\left(x_{i},y_{i}\right)\in V$ is uniformly sampled within free space. Each viewpoint is connected to its 20 nearest neighbors if the straight-line segment is collision-free, forming edges $e_{ij}=\left(v_{i},v_{j}\right)\in E$. The resulting graph G=(V,E) captures both local geometry and traversability.

Each vertex $v_{i}$ is associated with the following attributes: (1) relative position $\left(x_{i}-x_{c},y_{i}-y_{c}\right)$; (2) a binary visit indicator $b_{i}$; and (3) the number of observable frontier points $u_{i}$, which serves as a proxy for information gain. We define the edge weight in the navigation graph as

(12) $$e_{ij}=\left\{\begin{array}{cc} -D(v_{i},v_{j}), & \mathrm{if}\;\mathrm{the}\;\mathrm{segment}\;v_{i}v_{j}\;\mathrm{is}\;\mathrm{collision}\text{-}\mathrm{free},\\ -\infty , & \mathrm{otherwise}, \end{array}\right.$$

where $D(v_{i},v_{j})$ denotes the Euclidean distance between viewpoints $v_{i}$ and $v_{j}$. Accordingly, the system state at time *t* is represented by

(13) $$s_{t}=G_{t}=\left(V_{t},E_{t}\right).$$

When all information gains satisfy $u_{i}=0$, the exploration task is considered complete.

#### 3.3.2. Action Space Design

The action space consists of neighboring vertices directly connected to the current position $v_{c}$:

(14) $$A_{t}=\left\{v_{i}|\left(v_{c},v_{i}\right)\in E_{t}\right\}.$$

The policy network $\pi (\cdot |\theta_{\pi })$ selects an action $a_{t}=v_{i}^{*}$ corresponding to the next viewpoint to explore. This high-level decision is forwarded to a local navigation module that generates collision-free motion commands. A rolling replanning mechanism continuously re-evaluates the selected goal based on the updated state, ensuring responsive and real-time decision-making.

#### 3.3.3. Reward Function Design

The reward function guides the learning behavior of the SAC agent. To balance exploration completeness and motion efficiency, we define five reward components.

1. Exploration coverage reward $r_{e}$:
  (15) $$r_{e}=\left\{\begin{array}{cc} k(\eta_{t}-\eta_{t-1}), & \mathrm{if}\;\eta_{t}>\eta_{t-1},\\ -0.002, & \mathrm{otherwise}, \end{array}\right.$$
  where $\eta_{t}=S_{k}/S_{u}$ is the ratio of explored area to total area at time *t*.
2. Frontier information gain reward $r_{n}$:
  (16) $$r_{n}=N_{f},$$
  where $N_{f}$ is the number of newly observed frontier points.
3. Frontier distribution variation reward $r_{b}$:
  (17) $$r_{b}={∥c_{t}-c_{t-1}∥}_{2},$$
  where $c_{t}$ is the centroid of the frontier distribution at time *t*.
4. Path cost penalty $r_{d}$:
  (18) $$r_{d}=-∥O_{t}-O_{t-1}∥,$$
  which penalizes redundant motion.
5. Exploration completion reward $r_{f}$:
  (19) $$r_{f}=\left\{\begin{array}{cc} 20, & \mathrm{if}\;\mathrm{the}\;\mathrm{environment}\;\mathrm{is}\;\mathrm{fully}\;\mathrm{explored},\\ 0, & \mathrm{otherwise}. \end{array}\right.$$

The overall reward is defined as

(20) $$r=\alpha_{e}r_{e}+\alpha_{n}r_{n}+\alpha_{b}r_{b}+\alpha_{d}r_{d}+r_{f},$$

where $\alpha_{e}=1$, $\alpha_{n}=\frac{1}{60}$, $\alpha_{b}=\frac{1}{80}$, and $\alpha_{d}=\frac{1}{16}$.

The complete workflow of the proposed SAC-based active exploration algorithm is summarized in Algorithm 1. The algorithm integrates perception, high-level decision-making, motion execution, and policy optimization into a unified reinforcement learning pipeline.

**Algorithm 1** SAC-Based Active Exploration for Mobile Robots
**Require:** Initial local map $M_{0}$, initial pose $\left(x_{0},y_{0}\right)$, policy network $\pi (\cdot |\theta_{\pi })$, Q-network $Q(\cdot |\theta_{Q})$**Ensure:** Exploration trajectory $P^{*}$, final reconstructed map $M_{\mathrm{final}}$
1:Initialize environment state $s_{0}=G_{0}=\left(V_{0},E_{0}\right)$2:Initialize replay buffer D←∅3:
**repeat**
4:   Sample viewpoints $V_{t}$ from local map $M_{t}$5:   Construct topological graph $G_{t}=\left(V_{t},E_{t}\right)$ and compute vertex attributes $\left(x_{i},y_{i},b_{i},u_{i}\right)$6:   Select next target viewpoint via policy: $a_{t}=\pi \left(s_{t}|\theta_{\pi }\right)$7:   Plan local path $\mathrm{path}(v_{c}\to a_{t})$ and execute robot motion8:   Update map $M_{t+1}$ using new LiDAR observations9:   Rebuild topology $G_{t+1}$ and update state $s_{t+1}$10:  Compute reward $r_{t}=\alpha_{e}r_{e}+\alpha_{n}r_{n}+\alpha_{b}r_{b}+\alpha_{d}r_{d}+r_{f}$11:  Store transition $\left(s_{t},a_{t},r_{t},s_{t+1}\right)$ into buffer D12:  Sample a mini-batch from D and update:$$\begin{array}{c} \theta_{Q}\leftarrow \theta_{Q}-\eta_{Q}\nabla L_{Q}\left(\theta_{Q}\right),\;\theta_{\pi }\leftarrow \theta_{\pi }-\eta_{\pi }\nabla L_{\pi }\left(\theta_{\pi }\right) \end{array}$$13:  Soft update target network: $\theta_{Q}^{\prime }\leftarrow \tau \theta_{Q}+\left(1-\tau \right)\theta_{Q}^{\prime }$14:**until** $u_{i}=0$ for all $v_{i}\in V_{t}$15:**return** $P^{*}$, $M_{\mathrm{final}}$



As shown in Algorithm 1, the SAC-based active exploration algorithm enables mobile robots to perform adaptive exploration under online map updates. By incorporating a topology-aware state space, multi-objective reward shaping, and rolling replanning, the proposed approach improves coverage efficiency while reducing path redundancy.

#### 3.3.4. Spatiotemporal Feature Fusion and Policy Evaluation Network

**Figure 5:**
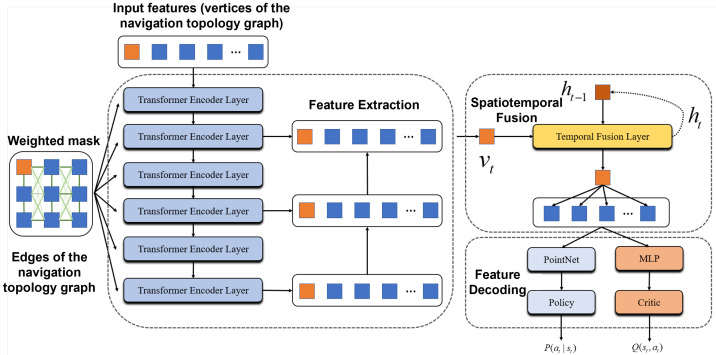
Architecture of the Spatiotemporal Policy Evaluation Network.

The proposed reinforcement learning framework employs a deep neural network to extract high-dimensional implicit representations from the explicit, low-dimensional attributes of the navigation topology graph, including vertex position, information gain, and connectivity. As illustrated in Figure 5, the network takes the vertices and edges of the navigation graph as input and outputs both the Actor policy and the Critic state–action value estimates for the current exploration state.

Specifically, the input graph is first processed by a six-layer Transformer-based encoder that aggregates features of adjacent nodes to capture local spatial correlations. A Node Pyramid Network (NPN) module is then applied to fuse multi-scale vertex features, enabling perception of structural information across different spatial resolutions. To model temporal dependencies, an LSTM-based module integrates memory vectors from previously visited nodes to produce a spatiotemporally fused feature representation of the current exploration state. Finally, a decoding network maps the fused representation to the action distribution over neighboring nodes for the Actor and to the corresponding value estimates for the Critic.

This hierarchical design enables the policy network to jointly reason about spatial topology and temporal exploration history, supporting long-horizon exploration decisions with improved efficiency and adaptability.

### 3.4. Coarse-to-Fine Multi-Resolution NDT Relocalization Based on Prior Map

To achieve robust and high-precision global relocalization during system initialization, we propose a coarse-to-fine multi-resolution Normal Distributions Transform (NDT) method based on a prior map. The initial pose inferred from BTC descriptor matching relies on sparse triangular-vertex correspondences; thus, limited geometric support and potential descriptor mismatches can yield large initialization errors. To refine this coarse estimate, the proposed method registers the current LiDAR scan to the prior map using a hierarchical multi-resolution probabilistic model, improving both accuracy and convergence robustness.

The core idea is to extend conventional NDT into a multi-resolution hierarchy inspired by pyramid-based image alignment. We construct NDT likelihood fields at multiple spatial resolutions and perform registration progressively from coarse to fine. At coarse levels, the likelihood field is more tolerant to initialization errors, enabling robust global alignment. As the resolution decreases, the pose is refined incrementally to achieve precise registration. This hierarchical strategy improves convergence robustness compared with single-resolution NDT and ICP-based approaches while maintaining high alignment accuracy.

In implementation, the target (prior) point cloud is voxelized at four resolution levels r={10m,5m,3m,1m} to construct multi-resolution NDT maps. For each voxel containing at least five points, we follow the standard NDT formulation [67] and model the local point set $\left\{p_{k}\right\}$ with a Gaussian distribution of mean μ and covariance Σ:

(21) $$\mu =\frac{1}{n}\sum_{k=1}^{n}p_{k},\;\Sigma =\frac{1}{n-1}\sum_{k=1}^{n}\left(p_{k}-\mu \right){\left(p_{k}-\mu \right)}^{T}.$$

Given a source point *p* transformed by a pose *T*, its likelihood in the corresponding target voxel is

(22) $$P\left(T,p\right)=\frac{1}{{\left(2\pi \right)}^{3/2}\sqrt{\left|\Sigma \right|}}\exp\;\left(-\frac{1}{2}{\left(Tp-\mu \right)}^{T}\Sigma^{-1}\left(Tp-\mu \right)\right),$$

which is the conventional NDT likelihood used for scan registration [52,67].

To improve robustness against outliers, we adopt a mixed Gaussian model consistent with robust NDT variants [67]:

(23) $$P\left(T,p\right)=c_{1}\exp\;\left(-\frac{1}{2}{\left(Tp-\mu \right)}^{T}\Sigma^{-1}\left(Tp-\mu \right)\right)+c_{2}P_{0},$$

where $c_{1}$ and $c_{2}$ are normalization coefficients determined by the probability integral constraint, and $P_{0}$ is a predefined noise ratio that mitigates the influence of outliers during optimization. A detailed derivation of the NDT-based registration objective and its optimization is provided in Appendix B.

**Figure 6:**
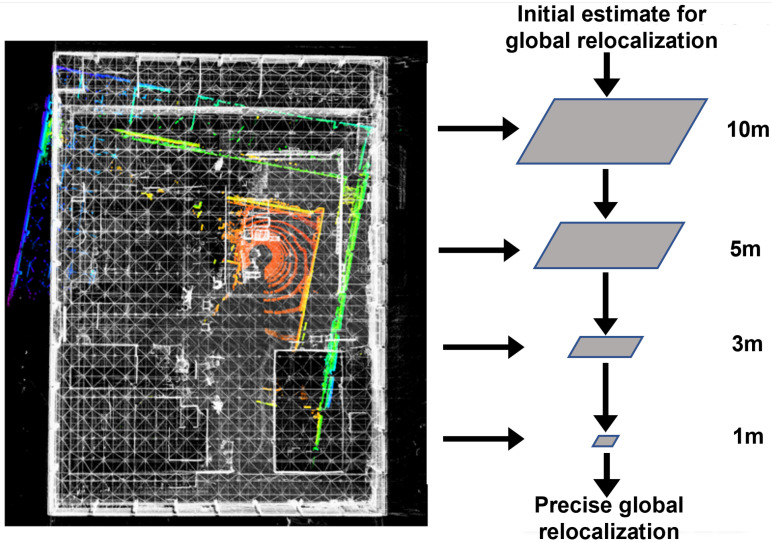
Multi-resolution NDT-based precise relocalization. The white point cloud represents the prior map, while the colored point cloud denotes the newly registered scan during relocalization. The proposed hierarchical optimization refines alignment accuracy progressively from coarse to fine resolutions.

On the basis of this probabilistic model, we employ the Levenberg–Marquardt (LM) algorithm to optimize the joint likelihood of all source points and estimate the transformation $T^{*}$ between the source and target point clouds. The optimization proceeds hierarchically from coarse to fine resolutions. As shown in Figure 6, the algorithm first performs a coarse alignment in the r=10m likelihood field using the BTC-based initialization and then successively refines the result in finer fields (r=5m,3m,1m), with each level initialized by the solution from the previous level. This hierarchical refinement promotes stable convergence toward an accurate global pose estimate.

Overall, the proposed coarse-to-fine multi-resolution NDT relocalization combines probabilistic modeling with hierarchical optimization to achieve precise and robust global pose estimation even under large initialization errors. This approach improves relocalization reliability in complex environments and provides a consistent initialization for multi-session SLAM and prior-map-based localization.

## 4. Experiments and Discussion

### 4.1. Experimental Platform

**Figure 7:**
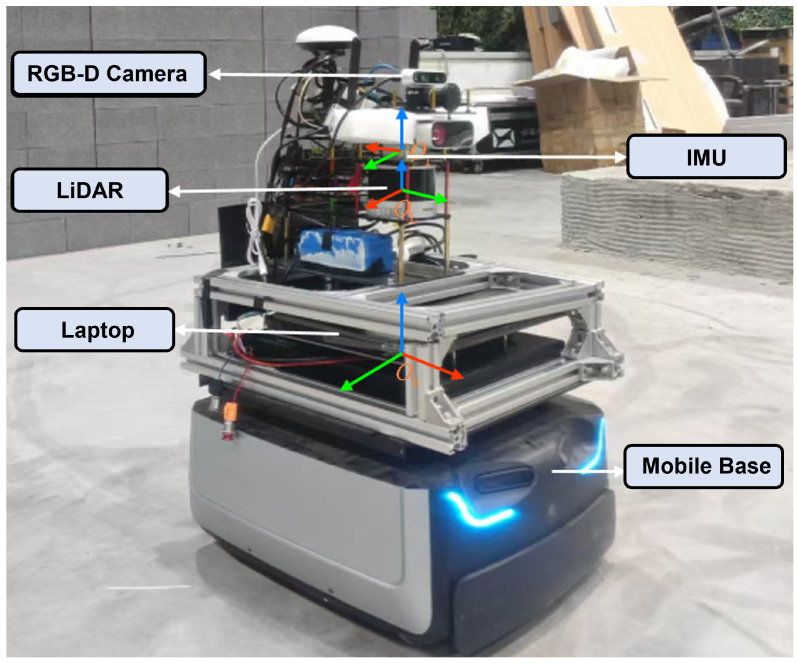
Mobile robot hardware platform and coordinate-frame definitions.

The mobile robot platform used in this study is shown in Figure 7. It is built on a differential-drive chassis and integrates multiple onboard sensors for real-time localization, mapping, and autonomous exploration. The robot is equipped with a 3D LiDAR, an inertial measurement unit (IMU), and an RGB-D camera, together with an onboard computing module for running the proposed algorithms. Although 3D LiDAR sensors may be relatively costly for small-scale applications, HV-LIOM targets research-grade and industrial mobile robots that require centimeter-level localization accuracy. The proposed adaptive hash-voxel representation reduces redundant memory usage and supports real-time operation on compact computing platforms such as Intel NUC and NVIDIA Jetson Xavier. The key hardware components and specifications are summarized in Table 1.

### 4.2. Real-Time Pose Estimation and Mapping

This section evaluates real-time pose estimation and point-cloud mapping performance based on the proposed adaptive hybrid hash-voxel algorithm. Experiments are conducted on the indoor Hilti dataset [68], the outdoor NCLT dataset [69], and our custom mobile robot platform to quantitatively assess accuracy and efficiency, and to qualitatively examine the reconstructed maps. For clarity, the hybrid voxel-based LiDAR–inertial odometry front-end is denoted as HV-LIO, while the SLAM system that integrates the adaptive hybrid hash-voxel representation with loop closure and global map optimization is denoted as HV-LIO-SAM. We compare our method with two representative LiDAR–inertial baselines, the optimization-based LIO-SAM [2] and the filtering-based FAST-LIO2 [3].

Pose estimation accuracy is evaluated using the Absolute Pose Error (APE) between the estimated trajectory and the ground truth. We report the maximum (Max), mean, median, root mean square error (RMSE), and standard deviation (Std). In addition, computational efficiency and map reconstruction quality are analyzed to provide complementary qualitative evidence.

#### 4.2.1. Indoor Experiment on the Hilti Dataset

The Hilti dataset is a benchmark for multimodal SLAM in complex indoor environments. It contains sparse-feature and repetitive-structure scenes and is well suited for evaluating LiDAR–inertial SLAM under challenging indoor conditions. Table 2 reports the APE RMSE of different algorithms across representative Hilti sequences. Both FAST-LIO2 and the proposed HV-LIO achieve centimeter-level accuracy on multiple sequences. Notably, HV-LIO consistently outperforms FAST-LIO2, indicating that the adaptive hybrid hash-voxel representation can improve data association stability and estimation accuracy in cluttered indoor scenes.

**Figure 8:**
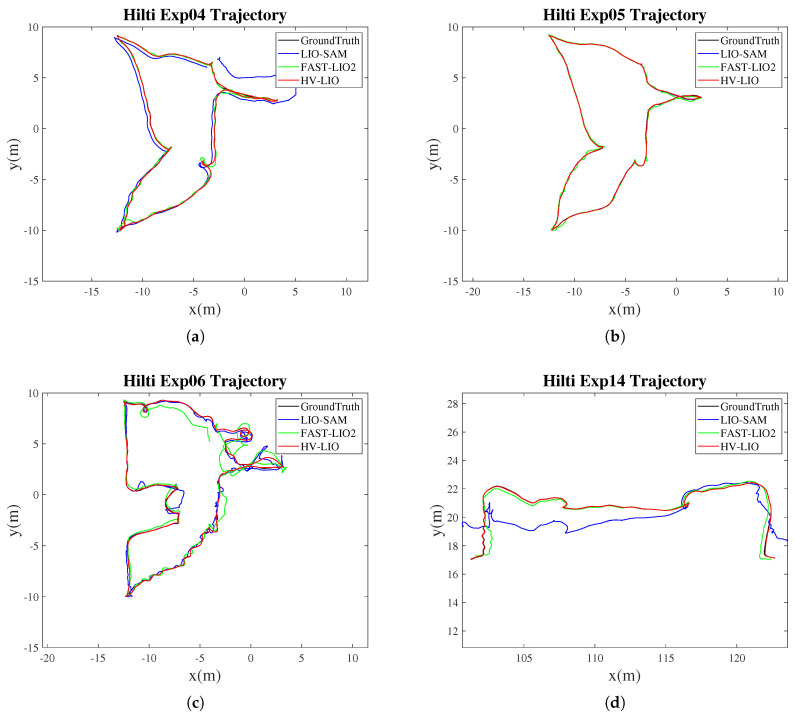
Trajectory comparisons on representative Hilti sequences. (**a**) exp04. (**b**) exp05. (**c**) exp06. (**d**) exp14.

Compared with FAST-LIO2, HV-LIO reduces the APE RMSE by approximately 5.7–17.3% across different sequences. A qualitative comparison between the estimated trajectories and the ground-truth trajectories is shown in Figure 8.

**Figure 9:**
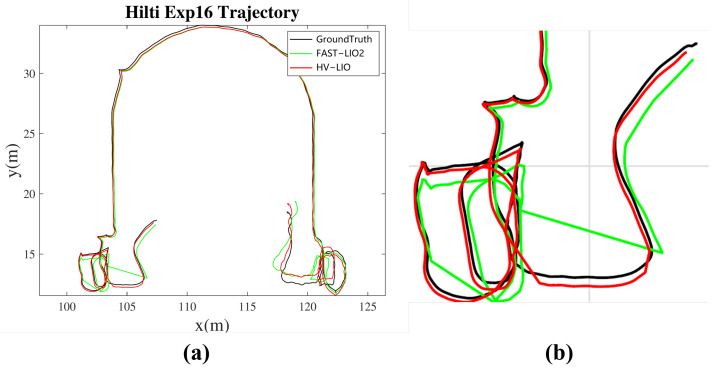
Trajectory comparison on the exp16 sequence of the Hilti indoor dataset. (**a**) Overall trajectories. (**b**) Zoomed-in local comparison.

To provide a more intuitive comparison, Figure 9 shows the overall and zoomed-in trajectories for the most challenging sequence, exp16. As shown in Figure 9b, the trajectory estimated by HV-LIO is closer to the ground truth than that of FAST-LIO2. As summarized in Table 2, HV-LIO achieves an RMSE of 0.3683 m on exp16, corresponding to a 15.2% improvement over FAST-LIO2 (0.4344 m). These results validate the effectiveness of the proposed adaptive hybrid hash-voxel representation for accurate indoor localization.

#### 4.2.2. Outdoor Experiment on the NCLT Dataset

**Figure 10:**
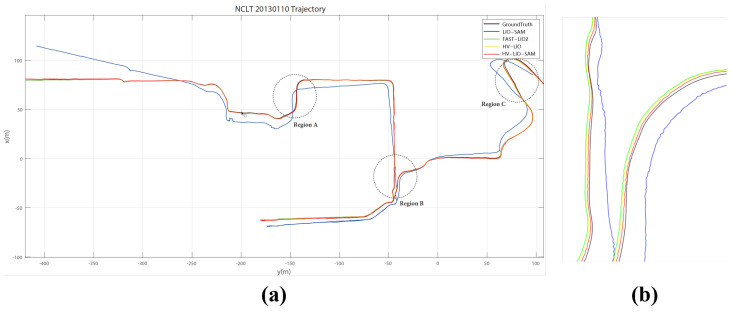
Trajectory comparison on the 0110 sequence of the NCLT dataset. (**a**) Overall trajectories. (**b**) Zoomed-in comparison in Region B.

The NCLT dataset is a large-scale, long-term benchmark for outdoor robotic localization and mapping. Figure 10a shows the estimated trajectories of HV-LIO, HV-LIO-SAM, LIO-SAM, and FAST-LIO2 on the 0110 sequence. The close-up view in Region B (Figure 10b) indicates that HV-LIO aligns more closely with the ground truth than FAST-LIO2. After integrating loop closure and global optimization, HV-LIO-SAM further improves trajectory consistency, demonstrating the benefit of the proposed back-end within the adaptive hash-voxel framework.

Quantitative APE statistics are summarized in Table 3. HV-LIO achieves a mean error of 0.77 m and an RMSE of 0.85 m, improving the RMSE by approximately 7.6% compared with FAST-LIO2. With loop closure and global optimization, HV-LIO-SAM further reduces the mean error to 0.64 m and the RMSE to 0.70 m, representing a 23.9% improvement over FAST-LIO2 and a 17.6% improvement over HV-LIO. These results confirm that the proposed loop-closure and pose-graph optimization effectively mitigate long-term drift in large-scale outdoor mapping.

#### 4.2.3. Mobile Robot Pose Estimation and Mapping Experiment

We further evaluate HV-LIO and HV-LIO-SAM on the physical mobile robot platform and compare their pose accuracy and mapping consistency with existing LiDAR–inertial methods. The experiment was conducted in a corridor environment in Building 6 of the National Defense Science and Technology Park at the Beijing Institute of Technology. The environment is narrow and contains long, repetitive corridors and multiple T-shaped junctions, posing challenges for LiDAR–inertial odometry and long-term map consistency.

**Figure 11:**
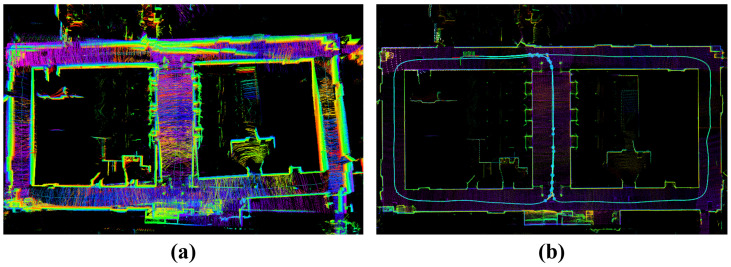
Point cloud mapping on the mobile robot platform. (**a**) FAST-LIO2. (**b**) Proposed HV-LIO-SAM.

Figure 11 compares FAST-LIO2 and HV-LIO-SAM in terms of reconstructed maps and estimated trajectories. The blue curve in Figure 11b denotes the robot trajectory, and the blue filled circles indicate loop closures detected by our system. These results suggest that the proposed learning-based loop-closure module can reliably identify revisited locations in repetitive corridor environments.

In regions visited only once, FAST-LIO2 produces visually consistent maps. However, in repeatedly traversed areas—particularly around the T-junctions—accumulated drift leads to visible misalignment and double contours, as shown in Figure 11a. By contrast, HV-LIO-SAM yields a more globally consistent map without obvious ghosting in revisited regions (Figure 11b). This indicates that combining the adaptive hash-voxel representation with loop closure and global pose-graph optimization improves map coherence in challenging indoor environments.

**Figure 12:**
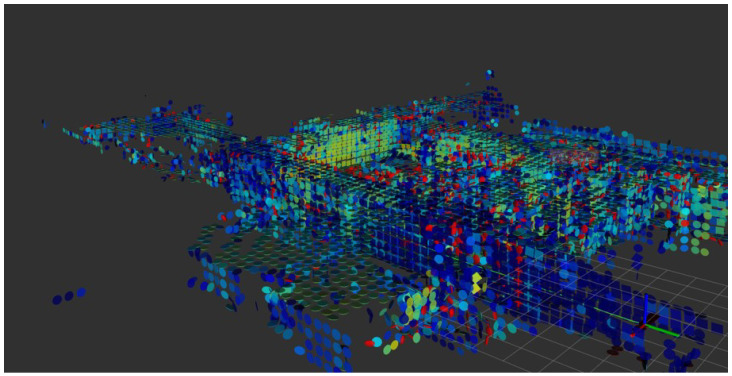
Voxel-level structural feature visualization of the proposed adaptive hybrid hash-voxel map.

To illustrate how the proposed map representation models scene structure, Figure 12 visualizes the voxel-level features built by HV-LIO-SAM. Circular patches correspond to locally fitted planar features, while square patches correspond to merged planar regions that represent extended structural planes such as walls or floors. Neighboring square patches form large continuous planes. Red ellipsoids denote non-planar features modeled as probabilistic volumetric Gaussians. Planar features are primarily associated with walls and the floor, whereas non-planar features tend to occur near structural edges such as wall intersections. These observations confirm that the adaptive hash-voxel map captures both planar and non-planar geometry and reuses these features as stable constraints during pose estimation.

We also compare trajectory accuracy among different methods in this environment. LIO-SAM, which relies on fixed edge and planar feature extraction, fails to maintain stable tracking and cannot complete the full trajectory. FAST-LIO2 produces a complete odometric trajectory via continuous-surface assumptions and incremental scan-to-map registration, but exhibits a maximum drift of 1.06 m along the vertical (*z*) axis. By contrast, HV-LIO and HV-LIO-SAM limit the maximum *z*-axis error to 0.32 m by leveraging the adaptive hybrid hash-voxel representation to better constrain local structure. This highlights the improved vertical stability of our method in indoor scenes where vertical observability is typically weak for ground robots.

We further benchmark runtime performance on an Intel(R) Xeon(R) E-2286M CPU @ 2.40 GHz to provide a hardware-independent reference. The per-frame timing breakdown is summarized in Table 4. For HV-LIO, the dominant components are (i) data preprocessing (point cloud downsampling, IESKF motion prediction, and deskewing), requiring 4.91 ms per frame on average; and (ii) data association and iterative state update within the IESKF, requiring 7.34 ms per frame. The overall average time per frame is 14.32 ms, comparable to FAST-LIO2 (13.65 ms). This corresponds to an effective pose-estimation rate of 69.83 Hz, which is higher than the 10 Hz scan rate of the LiDAR. These results demonstrate that the proposed method delivers accurate and drift-suppressed pose estimates in real time and is suitable for deployment on resource-constrained mobile robot platforms.

In addition to the quantitative comparisons in Section 4.2, a structured summary of methodological differences between HV-LIOM and representative state-of-the-art approaches is provided in Table A1 (Appendix A), highlighting the complementary advances in mapping, loop closure, prior-map relocalization, and autonomous exploration.

### 4.3. Active Exploration in Unknown Environments

**Figure 13:**
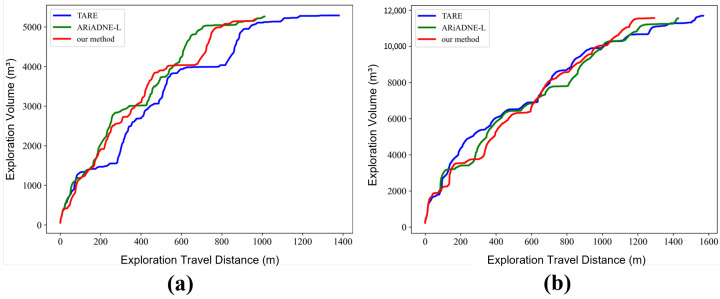
Relationship between exploration volume and travel distance for different algorithms. (**a**) Indoor Scene 1. (**b**) Indoor Scene 2.

This section validates the effectiveness and generalization of the proposed SAC-based active exploration algorithm in simulated environments. We compare our method with two representative 3D LiDAR-based autonomous exploration baselines: the sampling-based hierarchical planner TARE [70] proposed by Chao Cao et al. and the reinforcement-learning-driven method ARiADNE-L [71] proposed by Yuhong Cao et al. The relationship between explored volume and travel distance for the three methods is illustrated in Figure 13.

The results show that our method and the baselines achieve comparable performance in the early and mid stages of exploration. In the later stage, however, the proposed algorithm exhibits a faster growth of explored volume and completes full exploration with a shorter total travel distance. These observations suggest that the designed reward shaping, together with spatiotemporal feature modeling, enables the SAC policy to learn more effective long-horizon exploration behaviors than TARE and ARiADNE-L.

Quantitative comparisons of the total travel distance and time required to complete full mapping are summarized in Table 5. In Scene 1, our method completes exploration in 550.78 s with a travel distance of 970.52 m, reducing the travel distance and exploration time by 4.12% and 5.29%, respectively, compared with ARiADNE-L. In Scene 2, the proposed algorithm achieves full exploration with a travel distance of 1293.37 m in 775.39 s, corresponding to 9.38% and 7.20% reductions in distance and time over ARiADNE-L. Overall, these results indicate that the proposed SAC-based strategy achieves more efficient long-term exploration than both sampling-based and learning-based baselines in indoor simulated environments.

### 4.4. Prior Map-Based Localization and Navigation Experiments

**Figure 14:**
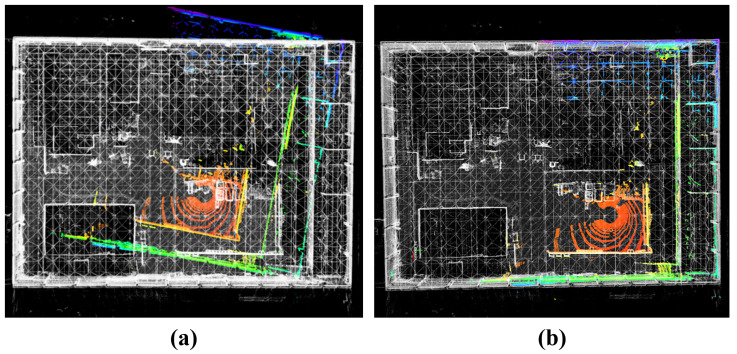
Point-cloud alignment before and after precise relocalization against the prior map. The colored point cloud denotes the registered scan, while the white point cloud represents the prior map. (**a**) Before relocalization (translation/rotation error: 10cm/$10^{\circ }$). (**b**) After relocalization.

To quantitatively evaluate the proposed multi-resolution Normal Distributions Transform (MR-NDT) relocalization, we align a LiDAR frame collected during autonomous mapping with the global prior point-cloud map. The reference pose for relocalization is taken as the estimated pose from the mapping session. To emulate initialization errors introduced by BTC-based global pose estimation, we apply random translational and rotational perturbations to the reference poses. A relocalization attempt is considered successful if the translation error is below 10 cm and the rotation error is below 3°. Four groups of experiments with different initial error magnitudes are conducted, each containing 100 random samples. The success rates of different methods are reported in Table 6, where bold and underlined entries denote the best and second-best results, respectively. A visual comparison of the point clouds before and after relocalization is shown in Figure 14.

As shown in Table 6, single-resolution NDT (e.g., NDT-3 at 3 m resolution) cannot simultaneously achieve robustness and accuracy across varying initialization errors. Coarser resolutions are more tolerant to large initial deviations but typically yield lower precision, whereas finer resolutions provide higher accuracy under small initial errors yet degrade rapidly as the initialization uncertainty increases. In contrast, the proposed MR-NDT integrates multiple resolutions in a coarse-to-fine scheme, thereby combining the complementary strengths of different scales. Even with initial translational and rotational errors of 10 m and 10°, respectively, MR-NDT achieves a 77% success rate, demonstrating strong robustness to large initialization uncertainty.

**Figure 15:**
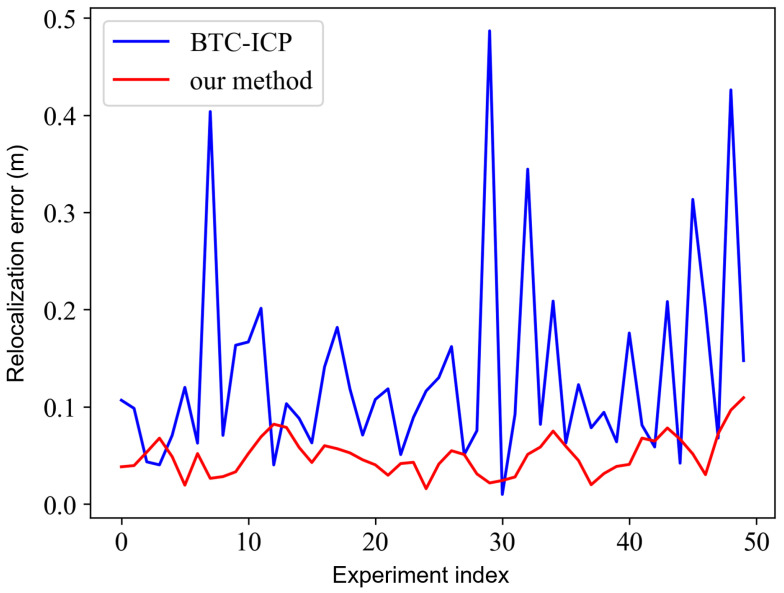
Accuracy comparison of global-initialization relocalization.

The relocalization error comparison between MR-NDT and BTC-ICP is shown in Figure 15. Across 50 independent trials, MR-NDT achieves an RMSE of 5.35 cm and a mean error of 4.97 cm, substantially outperforming BTC-ICP (RMSE 16.69 cm, mean 13.24 cm). Moreover, MR-NDT maintains a localization error below 10 cm in 48 out of 50 trials, indicating reliable accuracy across different initial positions and viewpoints.

#### 4.4.1. Real-Time Localization and Navigation Experiments

After the global initialization relocalization described in Section 4.4, we conduct real-time localization and navigation experiments using the estimated initial pose to validate prior-map-based deployment. In such scenarios, localization accuracy is more critical than incremental mapping accuracy: as long as the current LiDAR scan can be accurately registered to the prior map, the navigation system can reliably guide the robot to the desired target in both the digital map and the physical environment. Because ground-truth trajectories are unavailable in the real indoor factory scene, we use the keyframe poses from the autonomous mapping session as reference for quantitative evaluation.

**Figure 16:**
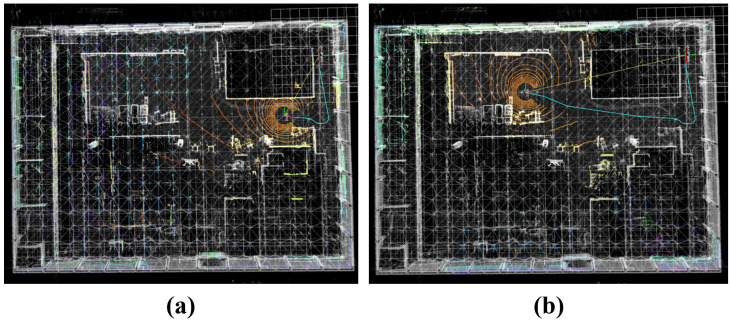
Real-time registration-based localization results: (**a**) at 65 s; (**b**) at 152 s. The real-time scan is aligned with the prior map.

As shown in Figure 16, two representative moments of the real-time localization process are visualized. The colored point clouds represent real-time LiDAR scans, while the white point cloud corresponds to the prior map constructed during autonomous exploration. The high degree of overlap indicates accurate scan-to-map alignment. The cyan curve denotes the estimated robot trajectory, suggesting that the proposed method provides stable and continuous localization during motion.

**Figure 17:**
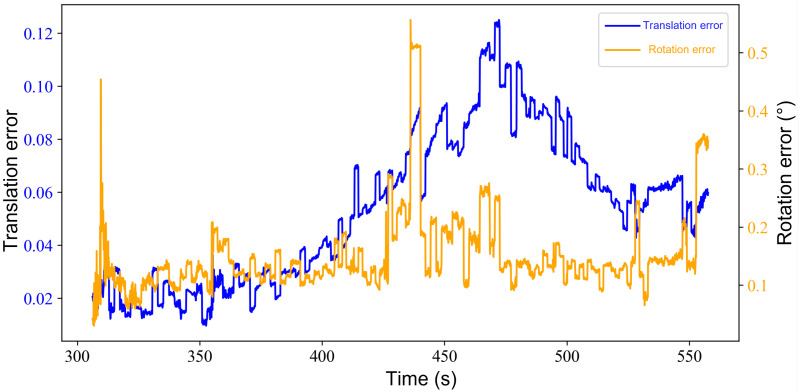
Translation and rotation error curves of the real-time localization experiment.

The translation and rotation error variations during real-time localization are shown in Figure 17. The rotation error remains nearly constant, and the translation error does not exhibit noticeable accumulation over time. This behavior is consistent with the proposed hybrid localization strategy, which combines a low-frequency, high-precision global registration module with a high-frequency local LiDAR–inertial odometry module to suppress long-term drift. Across all time steps, the mean translation error is 5.35 cm and the mean rotation error is 0.15°, meeting the precision requirements for indoor mobile-robot localization.

**Figure 18:**
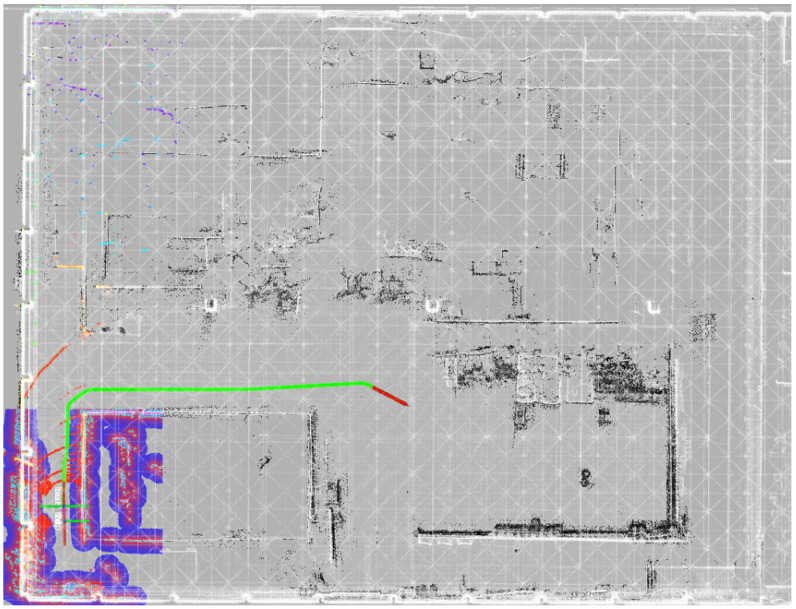
Autonomous navigation of the mobile robot based on a prior map.

Finally, autonomous navigation experiments are conducted using the prior point-cloud map, the derived 2D navigation grid map, and real-time localization information. As shown in Figure 18, the white point cloud represents the 3D prior map, the black region at the bottom indicates the 2D navigation grid map, and the colored points show real-time LiDAR perception. The red arrow denotes the navigation goal, and the green curve is the global path planned by the navigation module. These results demonstrate that the proposed system can integrate multi-source map representations, plan collision-free paths, and guide the robot to designated targets reliably.

#### 4.4.2. Ablation Study

To verify the effectiveness of the coarse-to-fine multi-resolution refinement, we conduct ablation experiments by removing specific resolution levels. As shown in Table 6, removing the coarsest 10 m level reduces the success rate from 77% to 26% under the 10 m–$10^{\circ }$ initialization error condition. Omitting the finest 1 m level also degrades performance, particularly under small-to-moderate initialization errors, indicating that fine-scale likelihood fields are important for precise convergence after coarse alignment. These results confirm that progressive multi-resolution refinement improves both convergence stability and alignment accuracy. Consistently, the post-relocalization scan aligns closely with the prior map in Figure 14b.

**Figure 19:**
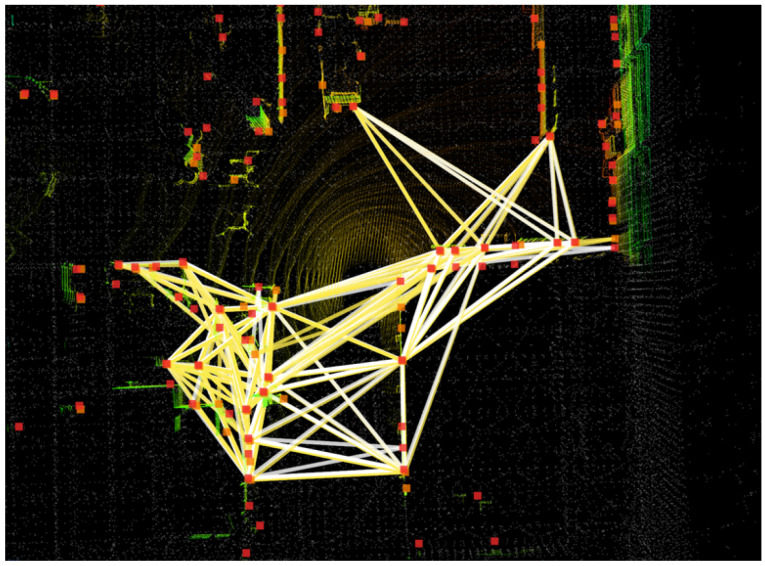
BTC keypoint detection and descriptor matching results.

To further assess global relocalization accuracy, we conduct 50 additional trials using keyframe point clouds from the mapping process without an external initial pose (identity initialization). We compare the absolute pose errors (APE) with those of BTC-ICP [65]. The corresponding BTC feature extraction and matching results are shown in Figure 19. Most extracted keypoints lie along walls, obstacles, and height-discontinuity edges, capturing informative local geometry. The matched BTC feature pairs (orange and red squares) and descriptors (white and yellow triangles) exhibit tight overlap, supporting the reliability of the proposed global initialization module.

In addition to voxel-resolution ablations, we evaluate the influence of key hyperparameters and network components in the SAC-based active exploration module. We focus on the learning rates ($\eta_{Q}$, $\eta_{\pi }$), discount factor (γ), and entropy temperature (α). The policy converges stably when γ is between 0.95 and 0.99 and α is between 0.05 and 0.2, consistent with typical SAC configurations. Excessively large α encourages over-exploration, whereas overly small α can reduce policy diversity and exploration efficiency.

We also analyze the impact of network architecture. Increasing the number of Transformer encoder layers from four to six improves spatial feature aggregation and long-horizon exploration success, while further increasing the depth yields marginal gains at higher computational cost. The LSTM-based temporal fusion module is critical for integrating sequential exploration history. Removing the LSTM reduces the coverage rate by approximately 6% and slows policy convergence, confirming the benefit of explicit temporal modeling for exploration.

Overall, these ablation results suggest that the proposed architecture provides a practical trade-off among learning stability, exploration efficiency, and computational cost, supporting reliable performance across diverse unknown environments.

## 5. Conclusions

This paper presents HV-LIOM, an integrated LiDAR–inertial SLAM and active exploration framework for efficient and robust 3D mapping in unknown environments. The proposed system comprises four key components: an adaptive hybrid hash-voxel map representation for efficient spatial management, a BTC-based loop-closure and global optimization module for long-term drift mitigation, a SAC-based active exploration policy that enables autonomous decision-making via spatiotemporal feature learning, and a multi-resolution NDT strategy for accurate prior-map-based relocalization.

Extensive experiments on public datasets and a custom mobile robot platform demonstrate the effectiveness of the proposed framework. On the Hilti indoor dataset, HV-LIO improves absolute pose accuracy by up to 15.2% over FAST-LIO2, and HV-LIO-SAM further enhances global consistency with loop-closure optimization. On the large-scale NCLT outdoor dataset, HV-LIO reduces localization RMSE by 7.6%, while HV-LIO-SAM achieves an additional 17.6% improvement after global optimization. For autonomous exploration, the SAC-based policy completes unknown-environment mapping with reduced travel distance and exploration time, achieving up to 9.4% and 7.2% reductions compared with representative sampling-based and learning-based baselines. For prior-map-based relocalization, MR-NDT attains an average RMSE of 5.35 cm, outperforming BTC-ICP by more than 68% and maintaining a 77% success rate under 10 m–$10^{\circ }$ initialization errors.

Overall, HV-LIOM improves accuracy, efficiency, and robustness across the mapping, loop-closure, relocalization, and exploration pipeline. By unifying adaptive voxelized mapping, learning-based loop closure, active exploration, and coarse-to-fine relocalization, this work advances LiDAR–inertial SLAM toward higher autonomy and long-term reliability in large-scale, complex environments.

Despite these advantages, LiDAR-based SLAM remains sensitive to adverse environmental conditions, including heavy rain, fog, strong sunlight, and dense vegetation, which can degrade range measurements and affect mapping quality. HV-LIOM is validated under standard indoor and outdoor conditions on the Hilti and NCLT datasets and demonstrates stable performance in typical scenarios. However, as the system primarily relies on geometric constraints from LiDAR and IMU, it may still face challenges in geometrically ambiguous scenes or environments with significant dynamic objects. Future work will investigate multimodal fusion with radar and RGB cameras and incorporate learning-based geometric priors to improve robustness and scene understanding in visually complex and adverse settings, with a particular focus on neural implicit mapping.

## Figures and Tables

**Table 1:** Hardware configuration of the mobile robot.

Component	Model/Parameters
Dimensions	0.54m×0.46m×0.82m
Drive type	Differential drive
CPU	AMD Ryzen 7 5800H @ 3.2GHz
LiDAR	Hesai PandarXT-32 @ 10Hz
IMU	LPMS-IG1 (9-axis AHRS)
RGB-D camera	Intel RealSense D435

**Table 2:** Comparison of absolute pose error (APE) RMSE (m) on the Hilti indoor dataset.

Method	exp04	exp05	exp06	exp14	exp16
LIO-SAM	0.8051	0.0627	0.8108	2.0336	*fail*
FAST-LIO2	0.0208	0.0209	0.0325	0.0367	0.4344
HV-LIO	**0.0172**	**0.0189**	**0.0298**	**0.0346**	**0.3683**

**Table 3:** Trajectory accuracy comparison on the NCLT dataset.

Method	Max (m)	Mean (m)	Median (m)	RMSE (m)
LIO-SAM	40.45	12.82	11.78	14.29
FAST-LIO2	2.05	0.87	0.79	0.92
HV-LIO	2.18	0.77	0.70	0.85
HV-LIO-SAM	**1.66**	**0.64**	**0.60**	**0.70**

**Table 4:** Comparison of the average computation time per frame for real-time pose estimation algorithms (ms).

Algorithm	Data Preprocessing	Data Association & Iterative Update	Map Incremental Update	Total Time per Frame
LIO-SAM	19.44	8.96	\	28.40
FAST-LIO2	4.86	8.27	0.52	13.65
HV-LIO	4.91	7.34	2.07	14.32

**Table 5:** Comparison of autonomous exploration performance in indoor simulated scenes.

Scene	Method	Exploration Distance (m)	Exploration Time (s)
Scene 1	TARE	1306.77	749.77
	ARiADNE-L	1012.24	581.57
	**HV-LIOM (SAC)**	**970.52**	**550.78**
Scene 2	TARE	1563.55	897.79
	ARiADNE-L	1427.35	835.59
	**HV-LIOM (SAC)**	**1293.37**	**775.39**

**Table 6:** Comparison of success rates (%) for precise relocalization methods under different initialization errors.

Method	1 m–1°	5 m–5°	10 m–10°	15 m–15°
NDT-3	43%	48%	8%	2%
MR-NDT (w/o 10 m)	99%	79%	26%	12%
MR-NDT (w/o 1 m)	44%	38%	42%	28%
**MR-NDT**	**99%**	**97%**	**77%**	**45%**

## Data Availability

The original contributions presented in this study are included in the article. Further inquiries can be directed to the corresponding author.

## Abbreviations

The following abbreviations are used in this manuscript:

HV-LIOM	Adaptive Hash-Voxel LiDAR–Inertial Odometry and Mapping (overall system)
HV-LIO	Hybrid Voxel-based LiDAR–Inertial Odometry (front-end odometry)
HV-LIO-SAM	Hybrid Voxel-based LiDAR–Inertial SLAM with Loop Closure and Mapping
SLAM	Simultaneous Localization and Mapping
LiDAR	Light Detection and Ranging
IMU	Inertial Measurement Unit
GNSS	Global Navigation Satellite System
LIO	LiDAR–Inertial Odometry
ICP	Iterative Closest Point
NDT	Normal Distributions Transform
SAC	Soft Actor–Critic
BTC	Binary Triangle Coding
APE	Absolute Pose Error
RMSE	Root Mean Square Error
BEV	Bird’s-Eye View
RGB-D	Red–Green–Blue and Depth
NCLT	North Campus Long-Term dataset

## Appendix A

**Table A1:** Structured comparison between HV-LIOM and representative LiDAR–inertial SLAM and localization methods.

Method	Map Representation	Loop Closure/Global Optimization	Prior-Map Relocalization	Autonomous Exploration	Main Characteristics
LOAM [1]	Feature-based (kd-tree)	Yes (pose graph)	No	No	High-accuracy LiDAR odometry with separate tracking and mapping threads.
LIO-SAM [2]	Feature-based (ikd-tree)	Yes (factor graph)	Limited (same-session)	No	Tightly coupled LiDAR–IMU optimization with keyframe-based loop closure.
FAST-LIO2 [3]	Direct mapping (ikd-tree)	No explicit loop closure	No	No	High-frequency direct LiDAR–IMU odometry with efficient incremental kd-tree.
LocNet [30]	Point-cloud/voxel features	No global exploration module	Yes (learned relocalization)	No	Deep learning-based pose regression or feature matching for global localization.
HV-LIOM (ours)	Adaptive hash-voxel with planar/non-planar probabilistic features	Yes: learning-based BTC loop closure + factor graph optimization	Yes: multi-resolution NDT-based prior-map relocalization	Yes: SAC-based active exploration	Unified framework for real-time SLAM, multi-session localization, and deep RL-driven exploration on a shared adaptive voxel map.

## Appendix B

In this appendix, we briefly outline the derivation of the NDT registration objective used in our multi-resolution relocalization module. Starting from the voxel-wise Gaussian model in (21) and (22), the negative log-likelihood of a source point *p* given pose *T* is

(A1) $$s\ell \left(T,p\right)=-\log P\left(T,p\right)=\frac{1}{2}{\left(Tp-\mu \right)}^{T}\Sigma^{-1}\left(Tp-\mu \right)+\mathrm{const}$$

Summing over all source points $\left\{p_{m}\right\}$ yields the overall objective

(A2) $$L\left(T\right)=\sum_{m}\ell \left(T,p_{m}\right)$$

which is minimized using a Levenberg–Marquardt (LM) optimizer on the SE(3) manifold, as described in Section 3.4. The robust mixture model in (23) is handled analogously by replacing P(T,p) in the negative log-likelihood.
